# Prevalence of Gastric Ulceration in Horses with Enterolithiasis Compared with Horses with Simple Large Intestinal Obstruction

**DOI:** 10.3390/vetsci9110587

**Published:** 2022-10-25

**Authors:** Valeria Albanese, Amelia Munsterman, Andreas Klohnen

**Affiliations:** 1 Tierärztliches Kompetenzzentrum für Pferde Großwallstadt Altano GmbH, 63868 Großwallstadt, Germany; 2 Department of Large Animal Clinical Sciences, Michigan State University, East Lansing, MI 48824, USA; 3 Chino Valley Equine Hospital, Chino Hills, CA 91709, USA

**Keywords:** horse, laparotomy, enterolithiasis, obstruction, colic, gastric ulcers, EGUS

## Abstract

**Simple Summary:**

Enteroliths are stone-like concretions of salts that form in the large colon of some horses. The disease associated with their presence is known as enterolithiasis and may manifest itself as chronic or acute abdominal pain. Enterolithiasis particularly occurs in some geographic areas such as California and Florida, although it has also been occasionally reported elsewhere. Gastric ulceration, defined as lesions of the mucosal lining of the stomach, is a well-documented pathological condition that affects horses of all ages and breeds throughout the world. Many risk factors for the development of gastric ulcerations have been identified, and some others have been suspected but never proven. This paper documents the prevalence of gastric ulceration in horses affected by enterolithiasis and were surgically treated and compares it with the prevalence of gastric ulceration in horses affected by similar intestinal diseases that were also surgically treated.

**Abstract:**

Enterolithiasis is a well-documented cause of colic in horses, especially in some geographic areas such as California and Florida. This retrospective case-control study aims at comparing the prevalence of gastric ulcers in horses affected by enterolithiasis to that in horses affected by other types of large intestinal obstruction. Two hundred and ninety-six horses were included in the study sample. Horses that had surgery for the removal of one or more enteroliths were included in the study as cases. Patients that had surgery for large intestinal simple obstructions other than enterolithiasis (large colon displacement, non-strangulating large colon torsion, and large and small colon impactions) were selected to match case horses for age, sex, and breed and included as controls. A total of 101/148 horses with enteroliths (68%) had gastric ulcers diagnosed during hospitalization, compared with 46/148 of matched controls (31%). There was a significant association between enterolithiasis and gastric ulceration (odds ratio 4.76, *p* < 0.0001), and a greater prevalence in Thoroughbreds as compared with other breeds (odds ratio 22.6, *p* < 0.0001). We concluded that enterolithiasis is significantly associated with gastric ulceration (*p* < 0.0001). The association is stronger in Thoroughbreds.

## 1. Introduction

Enterolithiasis is a well-known cause of colic due to partial or complete obstruction of the large intestine. It is reported worldwide, but more frequently in certain geographic areas such as California and Florida [1]. Enteroliths are formed in the right dorsal colon when ammonium phosphate crystals become arranged in concentric layers around a nidus [2]. Once formed, they can remain in the ascending colon or travel to the transverse and/or small colon. Horses with enteroliths in the large colon usually display recurrent mild-to-moderate signs of colic, whereas horses with enteroliths in the transverse or small colon have more acute and unrelenting pain, due to complete obstruction of the segment of the intestine where the enterolith(s) lodge. These horses are typically very gas distended, which may contribute to the signs of abdominal pain [3].

Gastric ulceration, also sometimes called Equine Gastric Ulceration Syndrome (EGUS) is a complex, multifactorial disease. Its pathogenesis is not completely understood, but several potential risk factors have been investigated and identified, including diet [4,5], breed [6,7,8,9,10], activity [6,7,8,9,11,12,13], stress [14,15], and NSAIDs administration [16,17,18,19,20,21].

Horses affected by gastric ulcers may be completely asymptomatic or they can display a variety of signs including acute and recurrent colic [22]; therefore, in many practices, a gastroscopy is considered a routine part of the diagnostic plan of horses presented with abdominal pain. Based on the authors’ observations of gastroscopies throughout the years, the impression was developed that horses with enteroliths might have a higher prevalence of gastric ulceration than horses that were presented with colic for reasons different than enteroliths; however, there was no evidence in the published literature to definitively support this claim. Pierce et al. 2010 [2] documented a 37–38% prevalence of gastric ulceration in horses affected by enterolithiasis, depending on the location of the obstruction. However, no controls were examined in that study.

The authors hypothesized that horses affected by enterolithiasis have a higher prevalence of gastric ulceration on presentation when compared with horses having large intestinal obstruction due to causes other than enteroliths. To prove this hypothesis, the authors compared the prevalence of gastric ulceration in horses with enterolithiasis to that in horses affected by other types of large intestinal obstruction.

## 2. Materials and Methods

Medical records from 10 consecutive years were reviewed for horses that underwent exploratory laparotomy and included in the study as cases if one or more enteroliths were found in surgery. In addition, these horses recovered from general anesthesia and had a gastroscopy of diagnostic quality performed during the first 24 h of hospitalization.

Moreover, during data collection, all horses admitted for signs of acute or chronic abdominal pain underwent a gastroscopy within 24 h from presentation, regardless of their clinical history and presenting signs, whenever it was safe and possible to do so. A gastroscopy was not performed on horses that were in uncontrollable pain, were extremely resentful of the procedure and difficult to handle, or when there was no consent from the owner.

The location of the enterolith or enteroliths was recorded as large colon, transverse colon, or small colon. It was recorded if the horse had enteroliths in more than one location, and those horses were included in the small colon or transverse colon groups (for example, if a horse had one enterolith in the large colon and one enterolith in the transverse colon, it was included in the transverse colon group. If a horse had one enterolith in the large colon and one enterolith in the small colon, that horse was included in the small colon group). There was no horse with enteroliths in both transverse and small colons.

Horses were excluded if factors considered as potentially confounding were present, namely: confirmed or suspected history of prolonged (meaning more than 24 h course) NSAIDs administration prior to hospitalization (not including perioperative NSAIDs use), active racing activity, concurrent small intestinal lesions found in surgery, postoperative incisional infections, prolonged postoperative pyrexia (meaning on more than one measurement), postoperative diarrhea, and postoperative nasogastric reflux of more than 2 L per hour retrieved from repeated intubation.

Horses were divided into groups based on breed, sex, and age.

Controls were selected amongst horses that had surgery in the same period for a large intestine simple obstruction other than enterolithiasis (large colon displacement, non-strangulating large colon torsion, and large and small colon impactions), recovered from general anesthesia, and had a gastroscopy performed within 24 h from admission. The same exclusion criteria were used for cases applied to controls. Controls were matched for the location of the lesion, the breed, and, when possible, for sex and age. If a control was not available in the same age group, a horse from the closest age group within the same breed was selected.

The presence or absence of gastric ulcers was recorded, and horses were classified as affected or non-affected.

The crude measure of association between the putative risk factor (enterolithiasis) and gastric ulceration was expressed as the odds ratio both for the group as a whole and for each different breed.

A chi-square test was used to determine the significance of the association between the presence or absence of enterolithiasis and the presence or absence of gastric ulceration, and to determine the significance of the association between the specific location of the enterolith and the presence or absence of gastric ulceration. Statistical significance was set at *p* < 0.05.

## 3. Results

A total of 1806 horses had an exploratory laparotomy performed during the time period considered. One hundred and forty-seven of them met the inclusion criteria for case horses. Among them, the breed distribution was as follows: 42 Arab or Arab cross (28.4%), 36 Quarter Horses (24.3%), 20 Thoroughbred (13.5%), 8 Andalusian (5.4%), 4 Miniature horses (2.7%), 3 Warmblood (2.1%), and 35 of other breeds (23.6%).

Sex was distributed as follows: 78 geldings (52.7%), 56 females (37.8%), and 14 stallions (9.5%).

Four groups were arbitrarily established as follows: horses up to 5 years old, horses between 6 and 12 years old, horses between 13 and 18 years old, and horses 19 years old or older. Age was distributed as follows: 5 horses were up to 5 years of age (3.4%), 52 were between 6 and 12 years old (35.1%), 50 were between 13 and 18 years old (33.8%) and 41 were 19 or older (27.7%) (mean: 14.66 ± 5.54, range 3–27).

One hundred fourteen cases (77%) had one or more enteroliths found in the large colon.

Thirteen cases (8.8%) had one enterolith found in the transverse colon. Of those 13, 6 (4.8% of all) had one or more enteroliths found in the large colon as well.

Twenty-one cases (14.2%) had one enterolith found in the small colon. Of those 21, 5 (3.4% of all) had one or more enteroliths found in the large colon as well.

Of the 148 non-enterolithiasis controls, 81 (54.7%) were geldings, 56 (37.8%) were females, and 11 (7.5%) were stallions.

A total of 4 horses were up to 5 years of age (2.7%), 53 were between 6 and 12 years old (35.8%), 52 were between 13 and 18 years old (35.1%) and 39 were 19 or older (26.4%) (mean: 14.76 ± 4.96, range 5–24).

One hundred fourteen controls had large colon simple obstructions (77%).

Thirty-four controls had small colon impactions (23%).

Out of 148 enterolithiasis cases, 101 (68%) had gastric ulcers diagnosed within 24 h from admission, as opposed to 46 out of 148 controls (31%). There was a significant association between enterolithiasis and gastric ulceration in our study (odds ratio 4.76, *p* < 0.0001).

The odds ratio was especially high in Thoroughbreds (22.6, *p* < 0.0001), as shown in Table 1.

Seventy-nine out of one hundred fourteen cases with large colon enteroliths had gastric ulcers (69.3%), as well as 9/13 with transverse colon enteroliths (69.2%) and 13/21 with small colon enteroliths (61.9%).

When a comparison was made according to the location of the enterolith(s), no significant difference in the prevalence of gastric ulceration was found between horses with large colon enteroliths and small colon enteroliths (*p* = 0.5), between horses with large colon enteroliths and transverse colon enteroliths (*p* > 0.5), and between horses with transverse and small colon enteroliths (*p* > 0.5).

In the control (non-enterolithiasis group), no significant difference in the prevalence of gastric ulceration was found between horses with small colon impactions (9/34, 26.5%) and large colon simple obstructions (37/114, 32.5%) (*p* = 0.5).

## 4. Discussion

The results of this study once again confirmed the widely reported high prevalence of gastric ulceration in domesticated adult horses [7,13,15,23,24,25,26,27,28,29,30].

The results of the study first reported an association between the presence of enterolith(s) and the presence of gastric ulcers. According to the statistical analysis, horses with enterolithiasis were almost five times more likely to have concurrent gastric ulcerations than horses affected by other types of simple large intestinal obstructions.

Several factors could contribute to gastric ulceration in the population examined, primarily due to stress from hospitalization in an unfamiliar environment, the presence of gastrointestinal disease, and fasting.

It is interesting to note that, despite comprehensive literature on equine gastric ulcers, comparatively little research has been conducted on the prevalence of this disease in hospitalized patients. Furr et al. [14] examined the detrimental effect of hospitalization on gastric ulceration in foals, and Rabuffo et al. [15] found that gastric ulcers worsened in 38/112 horses treated for colic during the initial five days of hospitalization. Stall confinement at a facility unfamiliar to the horses was one of the factors involved in an increased incidence of gastric ulceration in another study [29].

In human medicine, 75% to 100% of patients hospitalized in intensive care units develop abnormalities of the gastric mucosa [31,32,33,34,35] and samples of gastric juice test positive for blood in 35% to 100% of critically ill patients [36,37].

The concurrent presence of another major gastrointestinal disease could be a triggering or contributing factor for the development of gastric ulcers; however, it did not appear to be significantly correlated to a higher incidence of gastric ulceration when compared with a control population without gastrointestinal disease, in a previous study [15].

The effects of fasting on the development of gastric ulceration in horses have been extensively researched, and a positive association between fasting and the development of gastric ulcers has been established [38,39]. Fasting could play a role in the study population by two mechanisms: horses affected by gastrointestinal disease are often off feed prior to presentation [40], and the ones that undergo abdominal surgery are held off feed for a period that varies according to the individual necessities.

During hospitalization, gastrointestinal disease and fasting were common in cases and controls, it appears that cases are almost 5 times more likely to have gastric ulcers compared with controls.

A possible, although mostly speculative, explanation could be that the presence of enteroliths could chronically delay gastric emptying through neural and/or endocrine pathways. It is proven in physiology that the presence of content in the small intestine delays gastric emptying via the enterogastric reflex and the secretion of colecystokinine. In addition, there could be a similar regulatory mechanism between the large intestine and the stomach of horses for which the presence of a foreign body in the large intestine could delay gastric emptying. Gastric ulceration in the squamous mucosa is directly related to the degree and severity of gastric acid exposure, and gastric motility and the rate of gastric emptying have been implicated in causing ulceration [41]. In people with gastroesophageal reflux disease, acid clearance time and consequent acid exposure of the esophageal mucosa are inversely correlated to the rate of esophageal and gastric motility [42]. Delayed gastric emptying or decreased gastric motility could potentially increase the exposure of the squamous mucosa to gastric juice and other aggressive factors, leading to ulceration [41]. Enterolithiasis could also intermittently cause distention of the cecum and colon severe enough to compress the duodenum and cause intermittent outflow obstruction, which in turn could increase the exposure of gastric mucosa to gastric juice.

Horses with enterolithiasis often present with a history of going off their feed for some time prior to admission [40]. The prolonged fasting due to inappetence prior to presentation could cause a higher prevalence of gastric ulceration in these horses when compared with horses affected by other types of obstructions with a more acute onset.

Lastly, the association between gastric ulceration and enterolithiasis may be a consequence of similar management practices.

It is well established that feeding alfalfa hay is a risk factor for enterolithiasis [43,44]. Although feeding alfalfa seems to cause enterolithiasis, it might be protective against ulcers. Indeed, horses fed with a high protein, high calcium diet had fewer and less severe gastric ulcers [4,5]. It is, therefore, unlikely that the practice of feeding alfalfa hay may have caused the higher prevalence of gastric ulcers in our case horses.

Stall confinement without daily access to the pasture has been implicated as a risk factor for developing both gastric ulceration [45] and enterolithiasis [44,46,47]. The data on the turnout routine were not recorded for the population of horses included in our study, so the effects of this variable cannot be analyzed.

Strict exclusion criteria were applied with two purposes: first, to be as specific as possible in analyzing the association between enterolithiasis and gastric ulcers by excluding horses with other concurrent risk factors for ulcers. Second, to limit our population to cases that did not develop significant postoperative complications, all underwent a comparable postoperative period and were not hospitalized for a prolonged period.

Confirmed or suspected history of prolonged NSAIDs administration was considered an exclusion criterion because controversial evidence exists on whether NSAIDs cause gastric ulcers: some studies indicate that they do [16,17,18,48], while other studies demonstrate the opposite [9,12,27,49,50]. NSAIDs inhibit cyclooxygenases activity, therefore inhibiting prostaglandin synthesis [51], in turn decreasing mucosal blood flow and mucus production, and increasing HCl secretion [4,52,53,54].

In one study, NSAIDs administration was shown to cause ulcers in the glandular mucosa and to increase their severity in the non-glandular mucosa [48]. Thus, NSAIDs are thought to cause more severe ulcers in the glandular stomach mucosa because of their effect on prostaglandin inhibition [4]. NSAIDs administration was considered a potential confounding factor; therefore, we excluded horses with a known history of NSAIDs administration and horses with concurrent orthopedic problems that could have led to NSAIDs administration.

Active racing activity was considered an exclusion criterion because substantial evidence exists supporting the especially high prevalence of gastric ulcers in horses that are in active race training [6,13]. Strenuous exercise has been proven to induce gastric ulcers [7,8,9,12] and to increase the severity of the lesions [7,8,9,12].

Based on supporting evidence in human medicine of an elevated incidence of gastric ulceration in intensive care patients [31,37], we suspected that horses with more severe illness would be more likely to have ulcers. Therefore, postoperative complications such as postoperative pyrexia, postoperative diarrhea, and postoperative nasogastric reflux of more than 2 L per hour retrieved on repeated intubation were all considered as potential confounding factors and affected horses were excluded from our study, although limited studies have been conducted in equine medicine. Particularly, horses that developed nasogastric reflux during hospitalization were excluded since this factor was also noted as a possible cause of gastric erosions in the literature [55].

It is true that some complications, such as incisional infection and post-operative reflux, occur generally outside the timeframe when data on gastric ulceration were collected (within 24 h from admission), so it is unlikely that including those horses would have caused any bias in our results and conclusions. Nevertheless, the authors decided to exclude all significant postoperative complications regardless of the time of occurrence.

Concurrent small intestinal lesions found in surgery were considered as potential confounding factors that could decrease the specificity of our findings; in fact, horses with concurrent small intestinal lesions might have had reflux or endotoxemia which could have altered the occurrence of gastric ulceration. Therefore, these horses were also excluded.

Additional confounding factors that were adjusted for in this study included breed, sex, and age.

Whether an association exists between age and gastric ulcers is debated [7,9,11,27,30,56,57,58], whereas enterolithiasis rarely occurs in horses younger than 5 years of age [59].

The mean age of horses included in this study was 14.7 years, but that only includes horses that had surgery and survived anesthesia and does not necessarily reflect the mean age of the totality of horses with enterolithiasis that were presented to the hospital. Some older horses may have been euthanized due to short life expectancy or concurrent diseases, so the actual mean age of horses with enterolithiasis that were presented to the hospital in the timeframe covered by the study might have been higher.

Sex was found to be associated with gastric ulcers in some studies [9,11,30].

Regarding breed, Arabians, Arabian cross, and Miniature breeds were found to be predisposed to the development of enteroliths in several studies [44,46,47,59,60,61].

In this study, Arabians were overrepresented compared with the hospital population, whereas the distribution of the remaining breeds reflected it.

On the other hand, gastric ulceration seems to be especially prevalent in Thoroughbred racehorses, 70% to over 90% of which are affected [6,7,8,9,10]. A lower prevalence of gastric ulcers was found in Standardbred (44%, 63.3% of the actively racing ones) [13]. Active endurance horses were affected in 67% [62], western performance horses in 40% [28], and riding school horses in 11% [30] of cases.

However, the studies relating to Thoroughbreds and gastric ulcers have been conducted in athletic populations, therefore it is hard to draw a line between the potential effect of the breed and the potential effect of the strenuous exercise that these horses perform daily.

It is interesting to note that in our study, where only non-racing Thoroughbreds were included, Thoroughbreds were 22.6 times more likely to develop gastric ulcers when they were affected by enterolithiasis than when they were affected by other forms of large intestinal simple obstructions. This is a much higher ratio than any other breed considered. Nevertheless, the prevalence of gastric ulceration in control Thoroughbreds was as low as 11%, lower than most other breeds considered.

Our study has several limitations. The first limitation is its retrospective nature, which might have led to inhomogeneity in diagnostic procedures and protocols, and incomplete data.

A second limitation is that, although the data prove a higher prevalence of gastric ulcerations in cases vs. controls, the clinical significance of this observation may not be high, since gastric ulceration in horses can asymptomatically occur [22,41,63,64].

A third limitation is that, although the results of our study support the hypothesis that enterolithiasis is significantly associated with gastric ulceration (*p* < 0.0001), whether the correlation is causative remains unclear.

Prior to ours, there has been only one study that described concurrent enterolithiasis and gastric ulceration [2]. In that study, the authors reported ulcers in 37% of horses with large colon enteroliths and 38% of horses with small colon enteroliths. However, no controls were present in that study.

Based on our data, the prevalence of gastric ulceration in horses that had surgery for enterolithiasis is almost five times as much as in horses that had surgery for other types of simple obstruction of the large intestine. Since gastroscopy was not always performed immediately upon admission, it is unknown whether horses with ulcers had them already upon presentation, or rather, they developed them between presentation and gastroscopy.

## 5. Conclusions

This study proves that horses affected with enterolithiasis are very likely to have gastric ulceration as well, indeed much more than horses with other kinds of large intestinal disease. 

Based on this fact, it is recommended that all horses with enterolithiasis undergo a diagnostic gastroscopy or are treated presumptively for gastric ulcers.

## Figures and Tables

**Table 1:** Comparative distribution of gastric ulcers in cases and controls amongst different breeds.

Breed	Number and % of Case Horses with Ulcers	Number and % of Control Horses with Ulcers	Odds Ratio	χ^2^ *p*-Value	CI
ARAB OR ARAB CROSS	29/42 (69%)	15/42 (36%)	4.01	0.002	1.61–9.96
QUARTER HORSE	22/36 (61%)	10/36 (28%)	4.08	0.004	1.51–11.00
THOROUGHBRED	16/20 (80%)	3/20 (15%)	22.6	<0.0001	4.37–117.46
ANDALUSIAN	5/8 (62%)	2/8 (25%)	5	0.13	0.58–42.79
MINIATURE	3/4 (75%)	1/4 (45%)	9	0.15	0.36–220.92
WARMBLOOD	2/3 (66%)	0/3 (0%)	4	0.4	0.13–119.22
OTHER	23/35 (66%)	15/35 (43%)	2.55	0.05	0.97–6.72
**TOTAL**	**101/148 (68%)**	**46/148 (31%)**	4.76	<0.0001	2.91–7.78

## Data Availability

Preliminary data were presented at the ACVS symposium in 2013, at the SIVE symposium in 2013, and the IVECCS convention in 2013.
